# Telomere Length and Emotional and Behavioral Problems in Children from the Prospective Birth Cohort INfancia y Medio Ambiente (INMA) Study

**DOI:** 10.3390/children12070875

**Published:** 2025-07-02

**Authors:** Irene Campos-Sánchez, Eva María Navarrete-Muñoz, Josep Xavier Barber-Valles, Dries S. Martens, Isolina Riaño-Galán, Amaia Irizar, Sabrina Llop, Mónica Guxens, Cristina Rodríguez-Dehli, Izaro Babarro, Manuel Lozano, Martine Vrijheid, Tim Nawrot, Desirée Valera-Gran

**Affiliations:** 1 InTeO Research Group, Miguel Hernandez University, 03550 San Juan de Alicante, Spain; icampos@umh.es (I.C.-S.); dvalera@umh.es (D.V.-G.); 2 Institute for Health and Biomedical Research of Alicante (ISABIAL), 03010 Alicante, Spain; 3 Joint Research Unit, Statistical Methods in Health Sciences UMH-FISABIO, 03202 Elche, Spain; xbaber@umh.es; 4 Department of Statistics, Mathematics and Computer Science, Miguel Hernández University of Elche, 03202 Elche, Spain; 5 Centre of Environmental Sciences, Hasselt University, Agoralaan Gebouw D, BE-3590 Hasselt, Belgium; dries.martens@uhasselt.be (D.S.M.); tim.narrow@uhasselt.be (T.N.); 6 Central University Hospital of Asturias, Oviedo University, 33011 Oviedo, Spain; isolinariano@gmail.com; 7 Group of Environmental Epidemiology and Child Development, Biogipuzkoa Health Research Institute, 20014 San Sebastian, Spain; a-irizarloibide@euskadi.eus (A.I.); izaro.babarro@ehu.eus (I.B.); 8 Faculty of Medicine and Nursing, University of the Basque Country, 48940 Leioa, Spain; 9 CIBER Epidemiology and Public Health (CIBERESP), Carlos III Health Institute, 28029 Madrid, Spain; sabrina.llop@fisabio.es (S.L.); monica.guxens@isglobal.org (M.G.); manuel.lozano@uv.es (M.L.); martine.vrijheid@isglobal.org (M.V.); 10 Epidemiology and Environmental Health Joint Research Unit, FISABIO-Universitat Jaume I-Universitat de València, 46020 Valencia, Spain; 11 ISGlobal, 08036 Barcelona, Spain; 12 ICREA, 08010 Barcelona, Spain; 13 Pompeu Fabra University, 08002 Barcelona, Spain; 14 Department of Child and Adolescent Psychiatry/Psychology, Erasmus MC, University Medical Centre, 3015 GD Rotterdam, The Netherlands; 15 Pediatrics Service, Central University Hospital of Asturias, 33011 Oviedo, Spain; crdehli@yahoo.es; 16 Department of Preventative Medicine, Food Sciences, Toxicology and Forensic Medicine Department, Universitat de València, 46100 Valencia, Spain

**Keywords:** telomere length, emotional problems, behavioral problems, internalizing problems, externalizing problems, childhood

## Abstract

**Background/Objectives**: This study aimed to examine the association between leukocyte telomere length (TL) measured at ages 4 and 8 and emotional and behavioral problems at age 8. We also explored whether changes in leukocyte TL between ages 4 and 8 were associated with outcomes. **Methods**: Data were obtained from a population-based birth cohort and included 647 children with TL at age 4 and emotional and behavioral assessments at age 8, 673 with TL and outcomes at age 8, and 315 with TL measured at both ages. TL was determined using quantitative PCR on blood samples and converted into z-scores for analysis. Emotional and behavioral problems—including internalizing, externalizing, and total difficulties—were assessed using the Strengths and Difficulties Questionnaire. Regression models were conducted using zero-inflated and negative binomial, adjusting for sociodemographic and lifestyle covariates. **Results**: No statistically significant associations were observed between leukocyte TL at ages 4 or 8, or TL changes over this period, and emotional and behavioral outcomes at age 8. **Conclusions**: Although no significant associations were found, further longitudinal research is warranted to clarify the role of TL as a potential psychobiomarker of emotional and behavioral disorders in childhood.

## 1. Introduction

Telomeres are nucleoprotein structures containing repeat sequences of DNA that cap the end of chromosomes and protect the cell’s genomic stability. However, with each cell division cycle, their length gradually shortens, leading to mitochondrial and metabolic dysfunction and cell cycle arrest [1,2]. Shortened telomere length (TL) has been linked to an increased risk of age-related diseases and premature mortality [3,4]. As a result, TL has been proposed as a biomarker to assess cellular damage accumulated throughout life [5]. Research on TL suggests that certain mechanisms linked to telomere shortening, including elevated cortisol levels, mitochondrial dysfunction, immune–inflammatory responses, and oxidative stress, may impact brain health by compromising neuronal survival, disrupting synapse formation and action potential generation, and contributing to specific regional alterations in brain volume [6]. Given the involvement of these processes in stress-related mental health conditions, TL has been increasingly explored as a potential “psychobiomarker”—that is, a biological indicator of psychological functioning and vulnerability to emotional and behavioral problems [7].

Internalizing and externalizing psychological symptoms are used to categorize two types of behavioral, emotional, and social problems. Internalizing problems refer to symptoms that are directed inward that primarily cause distress to the child, such as anxiety, depression, somatic complaints, and withdrawal [8]. In contrast, externalizing problems encompass outward-directed symptoms that not only affect the child but also create discomfort for others [9], including aggressive and oppositional behaviors, inattention/hyperactivity, and emotion dysregulation [9]. A recent meta-analysis involving children aged 1–7 years from eight countries estimated an overall prevalence of mental disorders at 20.1%. Specifically, the prevalence rates for anxiety disorders, depressive disorders, oppositional defiant disorder, and attention-deficit hyperactivity disorder were 8.5%, 1.1%, 4.9%, and 4.3%, respectively [10].

Previous observational studies and meta-analyses conducted with adults and adolescents have established a connection between TL and internalizing psychological symptoms, including depression, anxiety, and post-traumatic stress disorder [11,12,13]. These investigations, conducted in both clinical and community samples, consistently reported that individuals with higher depressive and anxiety symptoms and disorders have shorter TL compared to individuals without such symptomatology [11,12,13]. In contrast, research on the association between TL and emotional and behavioral problems in the pediatric population remains limited. Only a few studies have explored this association, with two reporting preliminary evidence of shorter telomeres associated with higher internalizing problems in children aged 5–6 years [14] and 8–10 years [15]. Moreover, other studies have identified associations between shorter TL and poorer externalizing behaviors in children aged 5–12 years [16] and oppositional defiant behavior in children aged 3–5 years [17].

Both internalizing and externalizing problems during childhood have been identified as potential predictors of negative behavioral, emotional, cognitive, and physical health outcomes in adolescence and adulthood [18]. In addition, these problems can disrupt learning processes and are correlated with poor academic performance [19]. Given the high prevalence of internalizing and externalizing problems at early ages and their significant impact on global health across childhood, adolescence, and adulthood, it is crucial to investigate the potential role of TL as a psychobiomarker in children. More importantly, research should also focus on changes in TL over time, as these dynamics may provide valuable insights into the cumulative effects of stress and adversity experienced during childhood. Understanding variations in TL could help elucidate the biological impact of psychosocial factors and highlight opportunities for early interventions to mitigate long-term health risks.

Based on previous studies showing that shorter telomere length is associated with a greater risk of internalizing and externalizing psychological symptoms, we hypothesized that shorter leukocyte TL at ages 4 and 8 would predict higher emotional and behavioral problems at age 8. Additionally, we hypothesized that greater reductions in TL between ages 4 and 8 would be associated with worse emotional and behavioral outcomes. Therefore, the objectives of this study were as follows: (1) to explore the association between leukocyte TL at 4 years and emotional and behavioral problems at 8 years; (2) to explore the association between leukocyte TL at 8 years and emotional and behavioral problems at 8 years; and (3) to explore the association between the change between leukocyte TL at 4–8 years and emotional and behavioral problems at 8 years.

## 2. Materials and Methods

### 2.1. Study Design and Population

This study used data from the INfancia y Medio Ambiente (INMA) project (https://www.proyectoinma.org/), a multicenter population-based Spanish birth cohort study. The INMA project includes mother–child pairs recruited from seven regions across Spain: Ribera d’Ebre, Menorca, Granada, Valencia, Asturias, Gipuzkoa, and Sabadell. Enrollments occurred during the first prenatal visit, specifically between gestational weeks 10 and 13, at the primary public hospitals or health centers serving each area. To be included in this study, mothers had to meet several eligibility criteria: they needed to reside in one of the designated study regions, be aged 16 or older, be expecting a singleton pregnancy, not be undergoing assisted reproductive treatment, plan to give birth at the local reference hospital, and have no issues with communication [20].

For this analysis, we concentrated on data collected during the 4- and 8-year follow-up visits. These time points correspond to standardized follow-up assessments in the INMA cohort, during which extensive health, developmental, and emotional and behavioral data were systematically collected. Blood samples were obtained at both time points, allowing for TL determination under comparable conditions.

**Figure 1:**
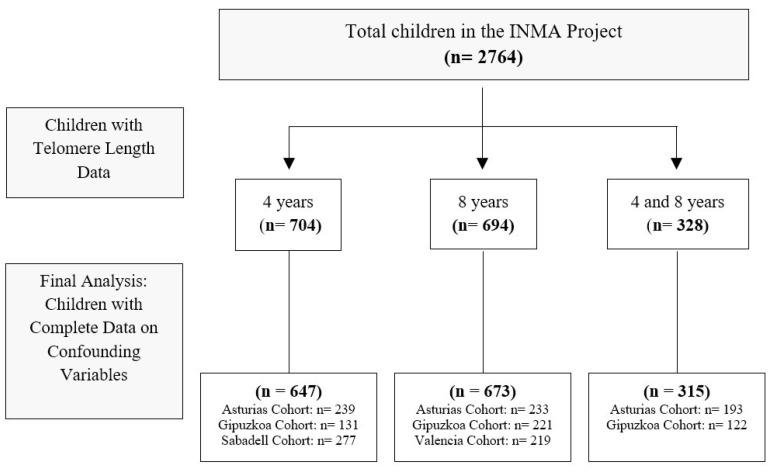
Flowchart of the study population from the INMA project.

A total of 647 mother–child pairs from Asturias, Gipuzkoa, and Sabadell cohorts were included, with data on leukocyte TL at 4 years and emotional and behavioral problems at 8 years. Additionally, 673 maternal–child dyads from Asturias, Gipuzkoa, and Valencia cohorts were included, with data on leukocyte TL and emotional and behavioral problems at 8 years. Lastly, 315 paired data from Asturias and Gipuzkoa with leukocyte TL measures at both ages were also incorporated. A detailed overview of the sample composition and data availability across cohorts is provided in the study flowchart (Figure 1).

All participants gave their informed consent upon enrollment in the INMA study. The research study protocol received approval from the ethics committee of the Miguel Hernández University of Elche (DPC.ENM.01.20).

### 2.2. Study Variables

#### 2.2.1. Telomere Length

Leukocyte TL data at 4 years were obtained for participants from the Asturias, Gipuzkoa, and Sabadell cohorts, while 8-year data were available for the Asturias, Gipuzkoa, and Valencia cohorts. TL was measured from the leukocyte fraction of blood samples collected at the corresponding follow-up visits, using a modified fluorochrome-based quantitative polymerase chain reaction (qPCR) protocol [21]. Each sample was analyzed in triplicate using a 7900HT real-time PCR system (Applied Biosystems, Waltham, MA, USA) with a 384-well plate format. To evaluate the efficiency of the qPCR reactions for both the telomeric (T) and single-copy gene (S) assays, a 6-point serial dilution was prepared from pooled DNA samples (n = 12) in every run. The resulting amplification efficiencies were 107% for the T assay (R^2^ = 0.994–0.999) and 97% for the S assay (R^2^ = 0.995–0.999). TL quantification was performed using qBase software [22] (Biogazelle, Zwijnarde, Belgium), and results were expressed as the ratio of telomere repeat copy number and single-copy gene quantity (T/S), standardized against the average T/S value of the complete dataset. To assess the precision of the measurement protocol, intraclass correlation coefficients (ICC) were computed for triplicate readings of telomeric (T) values (ICC = 0.957; 95% CI: 0.954–0.96; *p*  <  0.0001), single-copy gene (S) values (ICC = 0.968; 95% CI: 0.965–0.97; *p*  <  0.0001), and T/S ratios (ICC = 0.925; 95% CI: 0.918–0.93; *p*  <  0.0001), using the ICC calculation code provided by the Telomere Research Network [23]. Further details on TL measurement procedures, quality control, and distribution by cohort are provided in the Supplementary Material.

For association analyses, leukocyte TL measurements were transformed into z-scores. This transformation involved converting the telomere measurements to a distribution with a mean equal to zero and a standard deviation of one [24].

#### 2.2.2. Emotional and Behavioral Problems

Emotional and behavioral problems were assessed at age 8 using the Strengths and Difficulties Questionnaire (SDQ) [25]. In this study, emotional and behavioral problems were assessed using the Spanish parent-rated version of the SDQ, completed by the child’s main caregiver. This version has been adapted and validated in Spain based on data collected from both parents and teachers in a large sample of Spanish children (n = 595) [26]. The SDQ is a validated instrument intended for use with children aged 4 to 16 years. It comprises 25 items rated on a 3-point Likert scale (0 = not true; 1 = somewhat true; 2 = certainly true). The tool evaluates five domains: emotional symptoms, peer relationship problems, conduct issues, hyperactivity, and prosocial behavior. Scores from emotional and peer problems subscales were combined to calculate the internalizing problems score (range: 0–20 points), whereas the conduct problems and hyperactivity subscales were combined to calculate the externalizing problems score (range: 0–20). A total difficulties score (range: 0–40) was also calculated by summing the scores of all subscales except the prosocial behavior scale. Higher scores on the total and subscale scores indicate more severe emotional and behavioral problems. The parent-reported version of the SDQ has shown adequate psychometric properties, with a Cronbach’s alpha of 0.76 for the total difficulties score and values ranging from 0.58 to 0.77 for individual subscales [26].

#### 2.2.3. Covariates

To control for potential confounding factors, we also considered information on sociodemographic and lifestyle variables that could be associated with TL and the development of child emotional and behavioral problems, as previously described in the literature [27,28,29]. Maternal variables collected during pregnancy interviews included age (years), educational level (primary or lower and secondary or university), smoking during pregnancy (yes or no), and preconception body mass index (BMI, kg/m^2^). Child-related variables included sex (male or female), age (years), and BMI (kg/m^2^), which were collected during follow-up visits at 4 and 8 years of age.

### 2.3. Statistical Analysis

All statistical analyses were conducted using R software (version 4.4.1; R Foundation for Statistical Computing). A two-sided significance threshold was set at 0.05. The distribution of continuous variables was assessed using the Kolmogorov–Smirnov test with Lilliefors correction.

Descriptive statistics were used to summarize maternal and child characteristics across different follow-up periods, including pregnancy and visits at 4 and 8 years. Categorical variables were summarized using absolute and relative frequencies (%), while continuous variables not normally distributed were described by median and interquartile range (IQR). To explore the association between leukocyte TL z-scores and emotional and behavioral problems at different time points, zero-inflated negative binomial (ZINB) regression models or negative binomial (NB) regression models were used, depending on the distribution of the outcome variable [30]. The internalizing problems score, being a count variable with an excess of zeros, was analyzed using a ZINB model to estimate adjusted odds ratios (ORs) and incidence rate ratios (IRRs). For the externalizing problems score and the total emotional and behavioral problems score, NB models were employed to calculate IRRs. These estimates represent the change in risk of emotional and behavioral problems associated with a one-unit increase in the TL z-score, expressed as either ORs or IRRs depending on the model used.

Covariates associated with telomere length and emotional and behavioral problems in bivariate analyses were selected and tested as potential confounders. Those that altered the main association by 10% or more were retained in the final models. Considering that biological aging may differ between boys and girls, we also tested for interaction between the child’s sex and the main outcomes. Statistically significant interactions were found between sex and telomere length at age 4 in relation to total emotional and behavioral problems (*p* = 0.011), and between sex and TL ranking change from ages 4 to 8 in relation to internalizing problems (*p* = 0.009). Consequently, we conducted stratified analyses by sex. A full set of interaction *p*-values is presented in the Supplementary Material. Cohort heterogeneity was assessed using the I^2^ statistic. To derive pooled estimates, we employed meta-analytic methods, applying fixed-effects models when heterogeneity was low (I^2^ < 50%) and switching to random-effects models in the presence of substantial heterogeneity (I^2^ > 50%).

## 3. Results

Table 1 summarizes the socio-demographic and lifestyle characteristics of the mothers and children across the three samples collected at various time points. Mother–child pairs were relatively equally distributed across cohorts, except for Gipuzkoa participants, who accounted for approximately two-thirds (61.3%) of the sample with complete data at both 4 and 8 years.

The median maternal age was 31–32 years, with over 80.0% of the mothers having attained secondary or university education. The median preconceptional BMI ranged from 22.7 to 22.9 kg/m^2^, and approximately three-quarters of the mothers abstained from smoking during pregnancy. Slightly more than half of the children were male across the three samples, with a median BMI of 16.7 kg/m^2^ at 4 years and 17.0 kg/m^2^ at 8 years.

Regarding the main variables, the median of leukocyte TL at 4 and 8 years (T/S ratio) was 1.0 (IQR = 0.9 to 1.2) and 1.0 (IQR = 0.8 to 1.1), respectively. The median difference in leukocyte TL between 4 and 8 years was 0.03 (IQR = −0.07 to 0.15). At 8 years, emotional and behavioral problems assessed using the SDQ showed a median internalizing problems score of 3.0 (IQR = 1.0 to 5.0) and a median externalizing problems score of 5.0 (IQR = 3.0 to 8.0). The total emotional and behavioral difficulties score had a median of 8.0 (IQR = 5.0 to 12.0), with a maximum possible score of 40.

The results of the association between leukocyte TL z-scores and emotional and behavioral problems (i.e., internalizing, externalizing, and total problems) in children from the INMA study are displayed in Table 2. Overall, no statistically significant associations were observed between leukocyte TL at 4 or 8 years, or TL ranking change from 4 to 8 years, and emotional and behavioral outcomes at age 8. Stratified models by sex (Table 3 and Table 4) also showed no significant associations in either girls or boys, and no clear sex-specific trends were identified. These findings suggest no consistent association between TL and emotional and behavioral problems in childhood, regardless of the age of measurement or sex.

## 4. Discussion

The present study explored the potential influence of leukocyte TL on emotional and behavioral symptoms at different time points during childhood, using data from a Spanish population-based birth cohort. No significant associations were found between TL and internalizing or externalizing problems in children.

The potential association between TL and increased risk of emotional and behavioral difficulties in childhood has been examined in other cohort studies, such as the study by Robinson and colleagues (2023) [16]. Using data from the European population-based HELIX exposome cohort, which included children from the INMA-Sabadell cohort, this study reported a significant association between a shorter TL and poorer externalizing behaviors in children at 6–11 years. The discrepancies between the findings from our study and those from HELIX may be partially explained by methodological differences. Notably, HELIX included a larger sample size (1159 children versus 673 in our study), likely providing greater statistical power to detect associations between TL and emotional and behavioral problems. Moreover, the two studies used different tools to measure emotional and behavioral problems: the Helix study employed the Child Behavior Checklist (CBCL), whereas the present study used the Strengths and Difficulties Questionnaire (SDQ). These tools assess different dimensions of emotional and behavioral functioning, which may partially explain discrepancies in results. Additionally, while the Helix study examined the association between TL and emotional and behavioral problems cross-sectionally at a single point (6–11 years), the present study adopted a longitudinal approach, including assessments at multiple time points (4 and 8 years) and analyzing changes in TL over time. This broader perspective offers unique insights but may also introduce complexities that could impact the detection of significant associations.

Similarly, a study conducted with a Bucharest cohort (n = 195) reported significant associations between shorter TL and higher scores of internalizing problems at 8–10 years, as well as shorter TL and greater scores in general psychopathology at 10–12 years [15]. This study used different methods to collect the key variables: TL was measured using buccal DNA, in contrast to blood DNA in this present study, and children’s risk of psychopathology was reported by teachers at 8–10 years and by both teachers and caregivers at 10–12 years using the MacArthur Health and Behavior Questionnaire. Furthermore, this study employed a longitudinal path analysis to examine the directionality of the association between TL and childhood risk of psychopathology, finding evidence of a bidirectional relationship. Specifically, shorter TL was associated with increased reporting of psychological symptoms, while internalizing problems at 8–10 years predicted shorter TL at 12–14 years. In contrast, the present study focused exclusively on TL as an exposure variable, examining its potential impact on emotional and behavioral problems over time, without assessing the reverse relationship. These findings suggest that while shorter TL may act as a biomarker for psychopathological symptoms, such symptoms may also contribute to accelerated TL shortening over time, potentially reflecting accumulative stress or adversity experienced during childhood.

Building on this bidirectional perspective, two additional studies have supported the potential impact of psychopathology on TL shortening in childhood. Kroenke and colleagues found an inverse association between internalizing problems and buccal TL in general population children aged 6–7 years from a San Francisco cohort [14]. Unlike the present study, this investigation examined the association inversely and had a smaller sample size (n = 78). It also used a different tool (MacArthur Health and Behavior Questionnaire) to assess psychopathological symptoms. Similarly, another study showed that children with oppositional defiant disorder at ages 3, 4, and 5 had shorter telomeres at ages 4 and 5 compared to children without this condition [17]. Together, these findings suggest a relationship between psychopathology and TL shortening. One proposed hypothesis is that biological processes associated with psychopathology, such as inflammation, oxidative stress, and dysregulated neuroendocrine functioning, may contribute to telomere erosion [31].

Evidence from adult populations consistently showed an association between psychopathology and shorter TL in cross-sectional studies, particularly with internalizing disorders such as depression [11,13]. However, the directionality of this association remains unclear. Mixed evidence exists, with some prospective research showing that psychiatric disorders may predict telomere shortening, while other studies indicate that short telomeres may predispose adults to psychopathology [12,32]. In the present study, we were unable to investigate the reverse association, as data on psychopathology prior to telomere measurements were not available.

Another plausible explanation for the lack of significant associations observed in our study could be related to the sample characteristics and limitations in statistical power. Specifically, the low prevalence of internalizing and externalizing problems at age 8 limited the number of cases available for analysis, thereby reducing the power to detect meaningful associations. In addition, TL measurements at both 4 and 8 years showed minimal variability. This restricted range may have further constrained our ability to detect significant associations, even if they exist. Moreover, it is important to consider that TL was measured at a relatively early developmental stage (age 4), for which limited comparative data exist. Telomere dynamics and their association with psychosocial exposures may manifest differently across developmental periods, and associations may be more evident later in life, when stress-related biological changes have accumulated. This may also contribute to the divergence between our findings and those of studies involving older populations.

Building on this consideration, the average scores reported on the SDQ scales suggest that the children included in our sample presented generally low levels of emotional and behavioral difficulties, consistent with a community-based cohort rather than a clinical population. This characteristic may have limited the variability in outcome measures and reduced the likelihood of detecting significant associations with TL. Nevertheless, this feature also strengthens the contribution of our findings to the existing literature, as most previous research linking telomere length and psychopathology has been conducted in clinical samples. By focusing on a non-clinical population, our study offers complementary evidence on the potential utility of TL as a biomarker across the spectrum of emotional and behavioral functioning in childhood.

This study presents several limitations that should be acknowledged. First, the measurement of TL was based on a qPCR protocol applied to blood samples. Although this method is widely used due to its cost-effectiveness and speed, it provides an average measurement across all samples and may be subject to measurement error. Second, while the regression models were adjusted for potential confounding variables, some degree of residual or unknown confounding may still be present. Third, the absence of significant associations in our study may also reflect limitations in measurement sensitivity or in the theoretical framework linking TL to child psychopathology. Although the SDQ is a widely used and validated screening tool, it may not capture subtle emotional and behavioral profiles that could be biologically linked to telomere dynamics. Likewise, the biological embedding of psychosocial stress through telomere attrition may be more complex and influenced by cumulative exposures, genetic susceptibility, or environmental buffering, which may not be detectable in mid-childhood alone. Future research should explore alternative explanatory pathways and consider combining telomere measures with other biomarkers and more specific assessments of child psychopathology.

Despite these limitations, this study also has notable strengths. It was conducted within the context of the INMA population-based cohort, which collected detailed and longitudinal information on numerous covariates from pregnancy through childhood, thereby reducing the risk of recall and selection bias. Moreover, the availability of TL measurements at two time points (ages 4 and 8) enabled a longitudinal approach to explore potential associations with emotional and behavioral symptoms. While these methodological features strengthen this study’s design, they do not necessarily ensure the detection of significant associations. Therefore, the findings should be interpreted with caution and considered exploratory in nature.

## 5. Conclusions

No significant associations were found between leukocyte TL at ages 4 and 8, or changes in TL over this period, and emotional and behavioral outcomes at age 8. While TL has been proposed as a psychobiomarker of chronic stress and early adversity, our findings suggest that its association with emotional and behavioral problems during childhood may be limited or context dependent. Further longitudinal research is needed to better understand the role of TL in child development, ideally extending follow-up into adolescence or early adulthood, using more sensitive and dimensional assessments of psychopathology, and integrating additional biological and environmental indicators of chronic stress.

## Figures and Tables

**Table 1:** Sociodemographic characteristics and lifestyles of mothers and children belonging to the INMA project (Spain).

Study Variables	Measures at 4 y (n = 647)	Measures at 8 y (n = 673)	Measures at Both 4 y and 8 y (n = 315)
Cohort, n (%)			
Asturias	240 (37.1)	234 (34.8)	193 (61.3)
Gipuzkoa	131 (20.2)	220 (32.7)	122 (38.7)
Sabadell	277 (42.7)	-	-
Valencia	-	219 (32.5)	-
Mother characteristics			
Age, median (IQR)	31.0 (29.0–34.0)	31.0 (29.0–34.0)	32.0 (30.0–35.0)
Educational Level, n (%)			
Primary or less	120 (18.5)	122 (18.1)	47 (14.9)
Secondary	274 (42.3)	273 (40.6)	125 (39.7)
University	254 (39.2)	278 (41.3)	143 (45.4)
Preconceptional BMI, median (IQR)	22.9 (20.8–25.6)	22.7 (20.7–25.6)	22.8 (20.6–25.3)
Smoking during pregnancy, n (%)			
Yes	166 (25.6)	200 (29.7)	81 (25.7)
No	482 (74.4)	473 (70.3)	234 (74.3)
Children characteristics			
Sex, n (%)			
Males	331 (51.1)	347 (51.6)	163 (51.7)
Females	317 (48.9)	326 (48.4)	152 (48.3)
BMI at 4 years, median (IQR)	15.9 (15.2–16.9)	-	16.1 (15.3–17.2)
BMI at 8 years, median (IQR)	16.7 (15.4–18.5)	17.0 (15.7–18.8)	17.0 (15.7–18.9)

BMI, body mass index; IQR, interquartile range; y, years.

**Table 2:** Association between leukocyte telomere length and emotional and behavioral problems (internalizing, externalizing, and global problems) in children of the INMA Project (Spain).

	Leukocyte Telomere Length								
	4 Years (n = 647) ^a^			8 Years (n = 673) ^b^			Change 4–8 Years (n = 315) ^c^		
Emotional and Behavioral Problems at 8 Years	Estimate (95% CI)	*p*-Value	I^2^ (%)	Estimate (95% CI)	*p*-Value	I^2^ (%)	Estimate (95% CI)	*p*-Value	I^2^ (%)
Internalizing problems ^a^									
Count (OR)	0.92 (0.54; 1.55)	0.753	34.1	0.72 (0.29; 1.82)	0.418	0.0	0.34 (0.06; 2.10)	0.263	0.0
IRR	1.02 (0.95; 1.09)	0.635	30.3	1.01 (0.95; 1.08)	0.663	0.0	1.06 (0.94; 1.19)	0.315	38.6
Externalizing problems (IRR)	0.97 (0.92; 1.03)	0.317	37.4	1.03 (0.99; 1.09)	0.146	4.0	0.98 (0.87; 1.11)	0.784	49.1
Total score (IRR)	0.99 (0.94; 1.03)	0.582	48.2	1.03 (0.98; 1.08)	0.232	30.3	0.98 (0.87; 1.11)	0.818	48.2

OR, odds ratio; IRR, incidence rate ratio. ^a^ Models adjusted by mother’s age (continuous), educational level (primary or less and secondary or university), smoking during pregnancy (yes or no), preconceptional body mass index (continuous), and the change values in body mass index and age between 4 and 8 years (continuous). ^b^ Models adjusted by mother’s age (continuous), educational level (primary or less and secondary or university), smoking during pregnancy (yes or no), preconceptional body mass index (continuous), child’s age (continuous), and body mass index at 8 years (continuous). ^c^ Models adjusted by mother’s age (continuous), educational level (primary or less and secondary or university), smoking during pregnancy (yes or no), preconceptional body mass index (continuous), the change values in body mass index and age between 4 and 8 years (continuous), and telomere length at 4 years (basal z-score).

**Table 3:** Association between leukocyte telomere length and emotional and behavioral problems (internalizing, externalizing, and global problems) in girls of the INMA Project (Spain).

	Leukocyte Telomere Length								
	4 Years (n = 317) ^a^			8 Years (n = 326) ^b^			Change 4–8 Years (n = 152) ^c^		
Emotional and Behavioral Problems at 8 Years	Estimate (95% CI)	*p*-Value	I^2^ (%)	Estimate (95% CI)	*p*-Value	I^2^ (%)	Estimate (95% CI)	*p*-Value	I^2^ (%)
Internalizing problems ^a^									
Count (OR)	0.88 (0.47; 1.63)	0.679	45.8	1.64 (0.60; 4.45)	0.330	33.1	0.87 (0.25; 3.00)	0.826	0.0
IRR	0.90 (0.70; 1.16)	0.414	82.0	1.08 (0.98; 1.19)	0.113	32.6	0.98 (0.84; 1.16)	0.880	47.0
Externalizing problems (IRR)	0.93 (0.86; 1.01)	0.093	38.0	1.05 (0.94; 1.20)	0.324	63.5	0.96 (0.84; 1.10)	0.565	43.4
Total score (IRR)	0.91 (0.78; 1.07)	0.261	78.3	1.07 (0.95; 1.21)	0.289	67.5	0.97 (0.80; 1.16)	0.720	63.7

OR, odds ratio; IRR, incidence rate ratio. ^a^ Models adjusted by mother’s age (continuous), educational level (primary or less and secondary or university), smoking during pregnancy (yes or no), preconceptional body mass index (continuous), and the change values in body mass index and age between 4 and 8 years (continuous). ^b^ Models adjusted by mother’s age (continuous), educational level (primary or less and secondary or university), smoking during pregnancy (yes or no), preconceptional body mass index (continuous), child’s age (continuous), and body mass index at 8 years (continuous). ^c^ Models adjusted by mother’s age (continuous), educational level (primary or less and secondary or university), smoking during pregnancy (yes or no), preconceptional body mass index (continuous), the change values in body mass index and age between 4 and 8 years (continuous), and telomere length at 4 years (basal z-score).

**Table 4:** Association between leukocyte telomere length and emotional and behavioral problems (internalizing, externalizing, and global problems) in boys of the INMA Project (Spain).

	Leukocyte Telomere Length								
	4 Years (n = 330) ^a^			8 Years (n = 347) ^b^			Change 4–8 Years (n = 163) ^c^		
Emotional and Behavioral Problems at 8 Years	Estimate (95% CI)	*p*-Value	I^2^ (%)	Estimate (95% CI)	*p*-Value	I^2^ (%)	Estimate (95% CI)	*p*-Value	I^2^ (%)
Internalizing problems ^a^									
Count (OR)	1.15 (0.73; 1.78)	0.541	0.0	0.85 (0.47; 1.52)	0.586	0.0	1.00 (0.48; 2.10)	0.999	0.0
IRR	1.01 (0.98; 1.20)	0.100	0.0	0.96 (0.88; 1.05)	0.416	0.0	1.10 (0.94; 1.31)	0.230	0.0
Externalizing problems (IRR)	1.04 (0.97; 1.11)	0.258	0.0	1.02 (0.96; 1.09)	0.444	0.0	1.01 (0.90; 1.14)	0.817	0.0
Total score (IRR)	1.06 (0.99; 1.13)	0.079	0.0	1.00 (0.94; 1.06)	0.936	0.0	1.05 (0.93; 1.17)	0.428	0.0

OR, odds ratio; IRR, incidence rate ratio. ^a^ Models adjusted by mother’s age (continuous), educational level (primary or less and secondary or university), smoking during pregnancy (yes or no), preconceptional body mass index (continuous), and the change values in body mass index and age between 4 and 8 years (continuous). ^b^ Models adjusted by mother’s age (continuous), educational level (primary or less and secondary or university), smoking during pregnancy (yes or no), preconceptional body mass index (continuous), child’s age (continuous), and body mass index at 8 years (continuous). ^c^ Models adjusted by mother’s age (continuous), educational level (primary or less and secondary or university), smoking during pregnancy (yes or no), preconceptional body mass index (continuous), the change values in body mass index and age between 4 and 8 years (continuous), and telomere length at 4 years (basal z-score).

## Data Availability

Access to the data is subject to ethical and legal restrictions established by the Ethics Committees of La Fe Hospital in Valencia and Miguel Hernández University. As stated in the informed consent provided to participants, we ensured the confidentiality of all personal information collected through questionnaires and related sources. Data access requests can be directed to the corresponding author, E.-M.N.-M., at enavarrete@umh.es.

## Supplementary Materials

The following supporting information can be downloaded at: https://www.mdpi.com/article/10.3390/children12070875/s1. Method S1: Average relative telomere length measurement using qPCR.; Figure S1: Distribution of leukocyte telomere length (T/S) in children at 4 years; Figure S2: Distribution of leukocyte telomere length (T/S) by cohort in children at 4 years; Figure S3: Distribution of z-score leukocyte telomere length (T/S) in children at 4 years; Figure S4: Distribution of z-score leukocyte telomere length (T/S) by cohort in children at 4 years; Figure S5: Distribution of leukocyte telomere length (T/S) in children at 8 years; Figure S6: Distribution of leukocyte telomere length (T/S) by cohort in children at 8 years; Figure S7: Distribution of z-score leukocyte telomere length (T/S) in children at 8 years; Figure S8: Distribution of z-score leukocyte telomere length (T/S) by cohort in children at 8 years; Figure S9: Distribution of leukocyte telomere length ranking change between 4 and 8 years; Figure S10: Distribution of leukocyte telomere length ranking change between 4 and 8 years by cohort; Figure S11: Distribution of z-score leukocyte telomere length ranking change between 4 and 8 years; Figure S12: Distribution of z-score leukocyte telomere length ranking change between 4 and 8 years by cohort; Figure S13: Correlation between leukocyte telomere length at 4 and 8 years; Table S1: Interaction between telomere length and child’s sex in relation to psychoemotional problems (internalizing, externalizing, and total score). Refs. [33,34].

## Abbreviations

The following abbreviations are used in this manuscript:

TL	telomere length
INMA	INfancia y Medio Ambiente (Childhood and Environment)
qPCR	quantitative polymerase chain reaction
T	telomere
S	single-copy gene
T/S	ratio of telomere copy number to the number of single-copy genes
ICC	intraclass correlation coefficients
SDQ	Strengths and Difficulties Questionnaire
BMI	body mass index
IQR	interquartile range
ZINB	zero-inflated negative binomial
NB	negative binomial
OR	odds ratio
IRR	incidence rate ratios
y	years
CBCL	Child Behavior Checklist
DNA	deoxyribonucleic acid
