# Supplementary material for: Telomere Length and Emotional and Behavioral Problems in Children from the Prospective Birth Cohort INfancia y Medio Ambiente (INMA) Study
Source: Children (Basel). 2025 Jul 2;12(7):875. doi: 10.3390/children12070875 (PMC12293159; doi:10.3390/children12070875)
Supplement: Supplementary file 1 [file children-12-00875-s001.zip › children-3724144-supplementary.pdf]

Supplementary Material

Section S1. Telomere Length Measurement

Methods S1. Average relative telomere length measurement using qPCR.

Average relative telomere length was measured using a modified qPCR protocol in accordance with Cawthon et al.1 that was reported by Martens and colleagues2. Firstly, DNA quantity and purity was assessed using a Nanodrop 1000 spectrophotometer (Isogen, Life Science, Belgium) considering the DNA pure when the A260/280 was greater than 1.80 and A260/230 greater than 2.0. DNA integrity was assessed by agarose gel-electrophoresis. To ensure a uniform DNA input of 5 ng for each qPCR reaction, samples were diluted and checked using the Quant-iT™ PicoGreen® dsDNA Assay Kit (Life Technologies, Europe). The samples All samples were measured in triplicate using a 7900HT Fast RealTime PCR System (Applied Biosystems) in a 384-well format. The reaction mixture used to assess telomeres contained 1x QuantiTect SYBR Green PCR master mix (Qiagen, Inc., Venlo, the Netherlands), 2 mM dithiothreitol (DTT), 300 nM telg primer (ACACTAAGGTTTGGGTTTGGGTTTGGGTTTGGGT TAGTGT) and 900 nM telc primer (TGTTAGGTATCCCTATCCCTATCCCTATCCCTATCCCTAACA). The applied cycling conditions were: 1 cycle at 95°C for 10 min, 2 cycles at 94°C for 15 sec and 49°C for 2 min, and 30 cycles at 94°C for 15 sec, 62°C for 20 sec, and 74°C for 1 min and 40 sec. The single-copy gene (human β globin) qPCR mixture contained 1x QuantiTect SYBR Green PCR master mix, 400 nM HBG1 primer (GCTTCTGACACAACTGTGTTCACTAGC) and 400 nM HBG2 primer (CACCAACTTCATCCACGTTCACC). The single-copy gene used in Sabadell samples at 8 years was different and contained 1x QuantiTect SYBR Green PCR master mix, 300 nM 36B4u primer (CAGCAAGTGGGAAGGTGTAATCC) and 500 nM 36B4d primer (CCCATTCTATCATCAACGG GTACAA)3. The same cycling conditions were used: 1 cycle at 95°C for 10 min, 40 cycles at 95°C for 15 sec, and 58°C for 1 min and 20 sec. After PCR cycling individual qPCR curves and melt curves were visually inspected and when a run error was observed the Cq value was removed from subsequent analysis. In addition, when triplicate measures showed a deviation of more than 0.3 in Cq value these were removed from subsequent analysis.

1. Cawthon, R.M. Telomere Length Measurement by a Novel Monochrome Multiplex Quantitative PCR Method. Nucleic Acids Res 2009, 37, e21, doi:10.1093/nar/gkn1027.

2. Martens, D.S.; Van Der Stukken, C.; Derom, C.; Thiery, E.; Bijnens, E.M.; Nawrot, T.S. Newborn Telomere Length Predicts Later Life Telomere Length: Tracking Telomere Length from Birth to Child- and Adulthood. EBioMedicine 2021, 63, 103164, doi:10.1016/j.ebiom.2020.103164.

3. Martens, D.S.; Janssen, B.G.; Bijnens, E.M.; Clemente, D.B.P.; Vineis, P.; Plusquin, M.; Nawrot, T.S. Association of Parental Socioeconomic Status and Newborn Telomere Length. JAMA Netw. Open 2020, 3, e204057, doi:10.1001/jamanetworkopen.2020.4057.

Section S2. Telomere Length Distribution

Figure S1. Distribution of leukocyte telomere length (T/S) in children at 4 years.

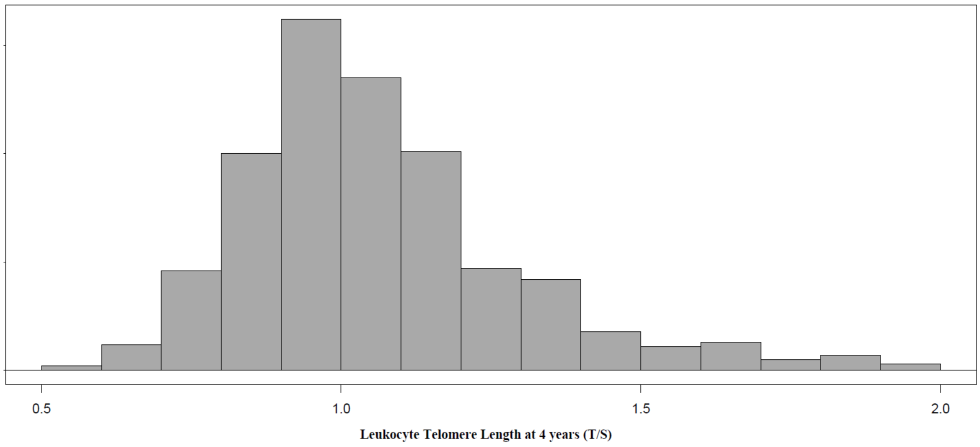

Figure S2. Distribution of leukocyte telomere length (T/S) by cohort in children at 4 years.

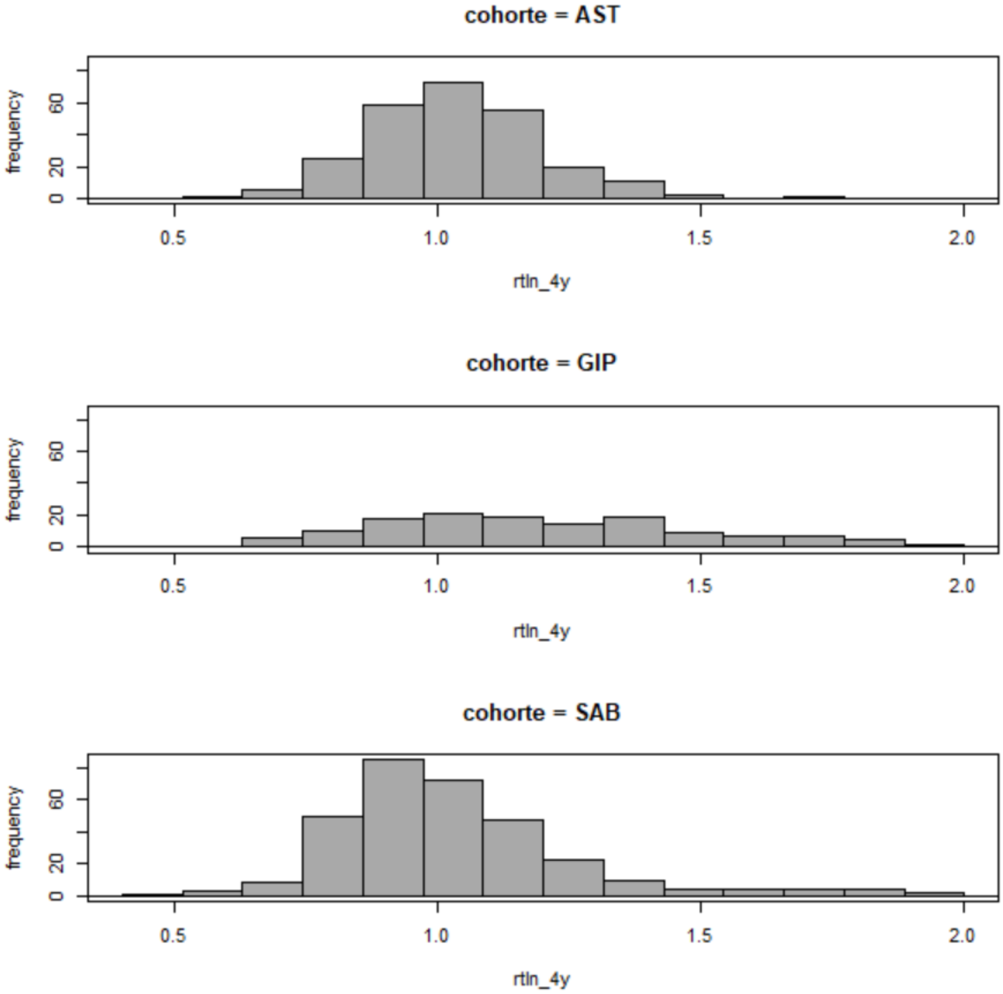

Figure S3. Distribution of z-score leukocyte telomere length (T/S) in children at 4 years.

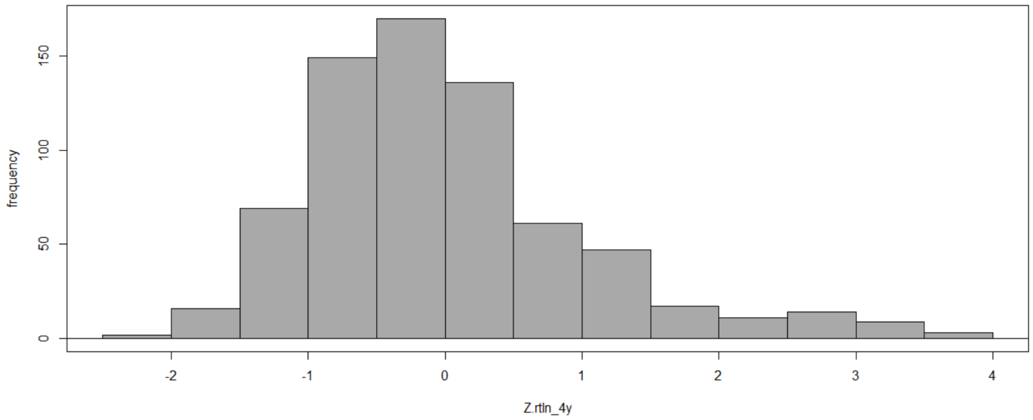

Figure S4. Distribution of z-score leukocyte telomere length (T/S) by cohort in children at 4 years.

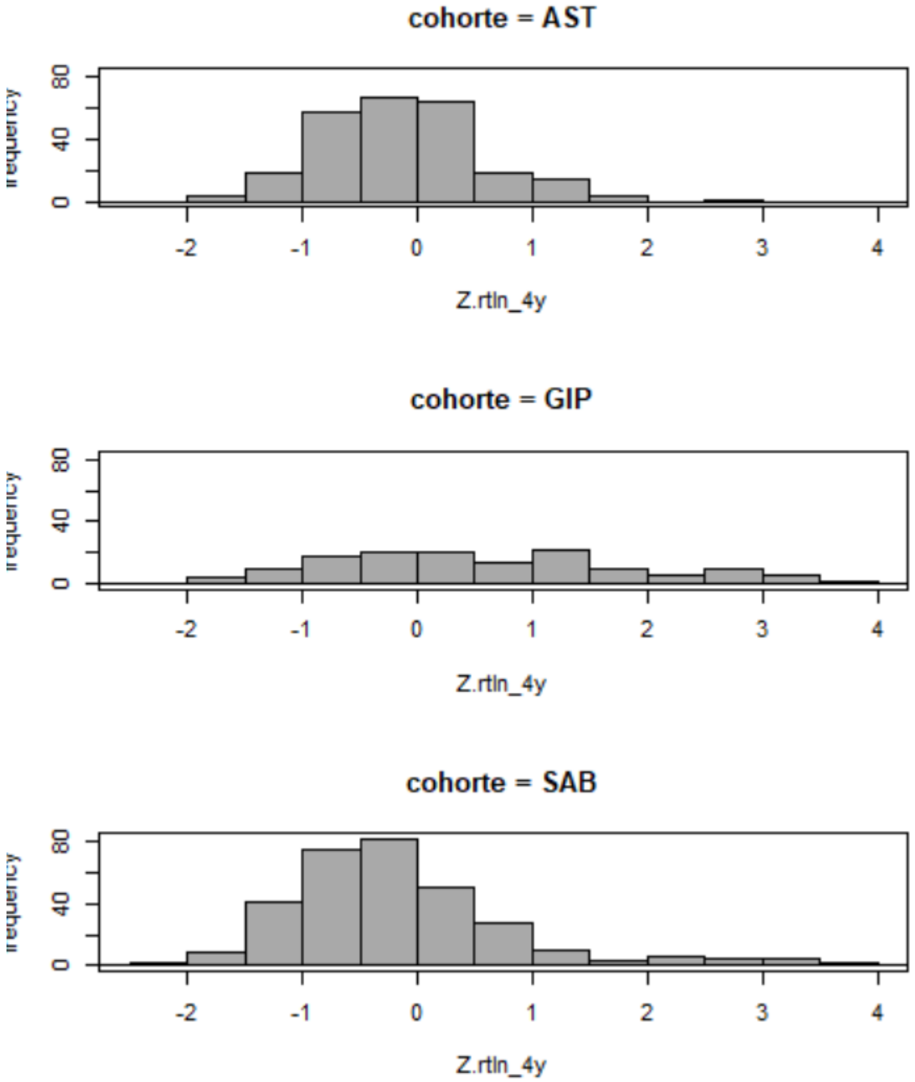

Figure S5. Distribution of leukocyte telomere length (T/S) in children at 8 years.

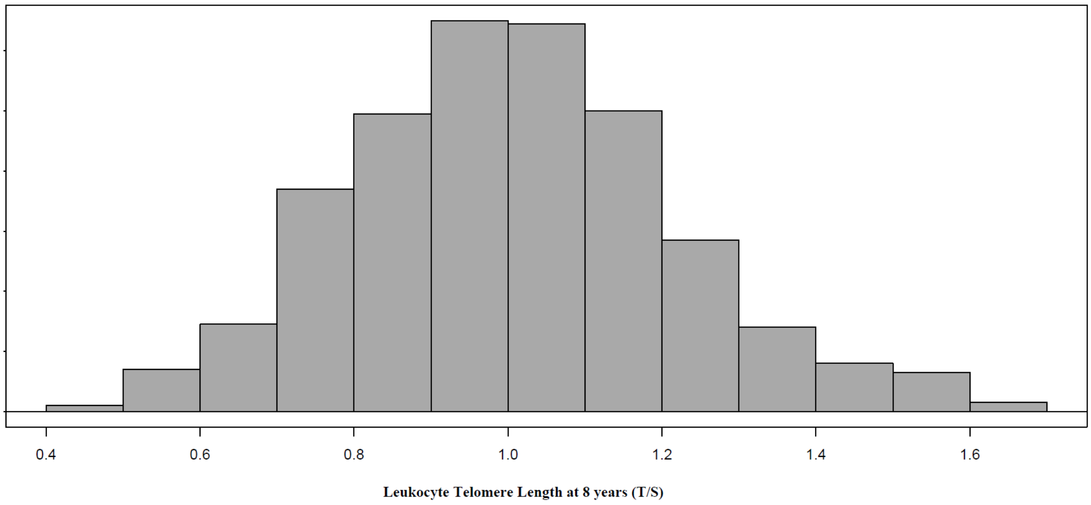

Figure S6. Distribution of leukocyte telomere length (T/S) by cohort in children at 8 years.

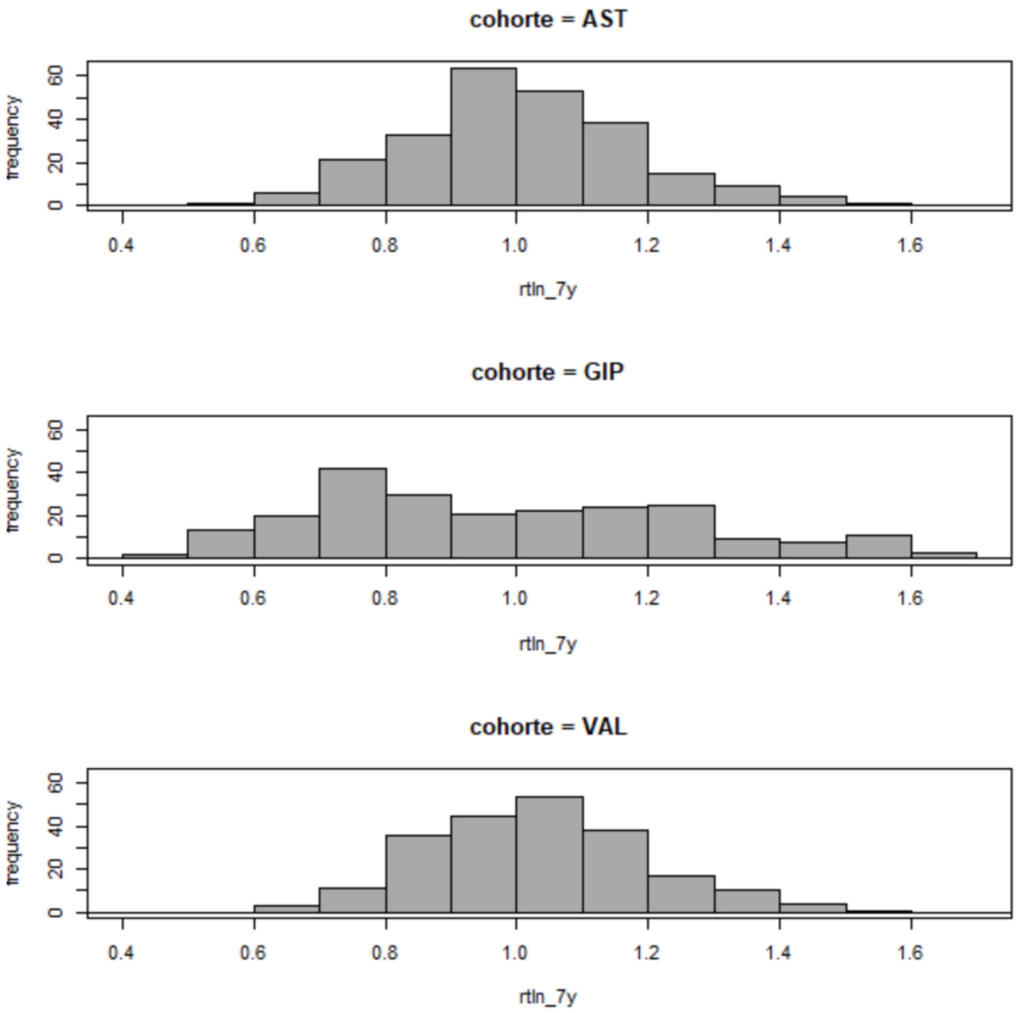

Figure S7. Distribution of z-score leukocyte telomere length (T/S) in children at 8 years.

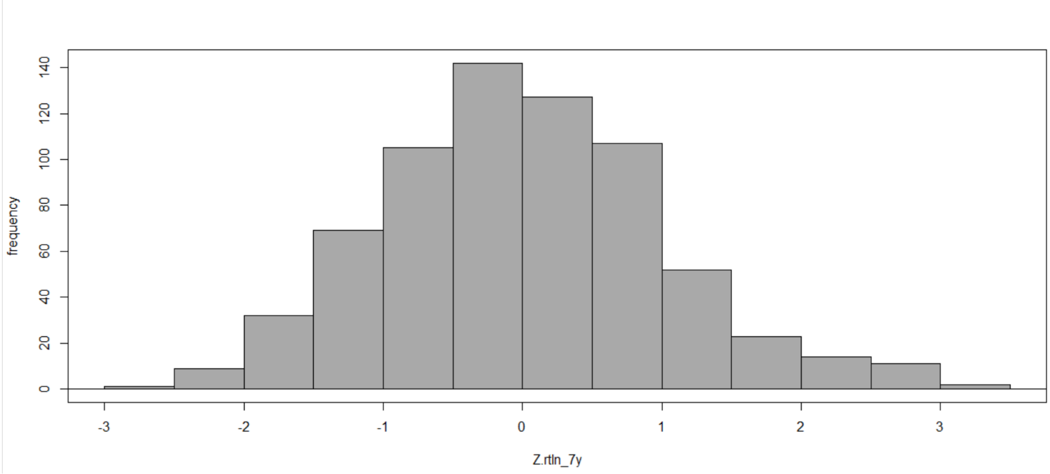

Figure S8. Distribution of z-score leukocyte telomere length (T/S) by cohort in children at 8 years.

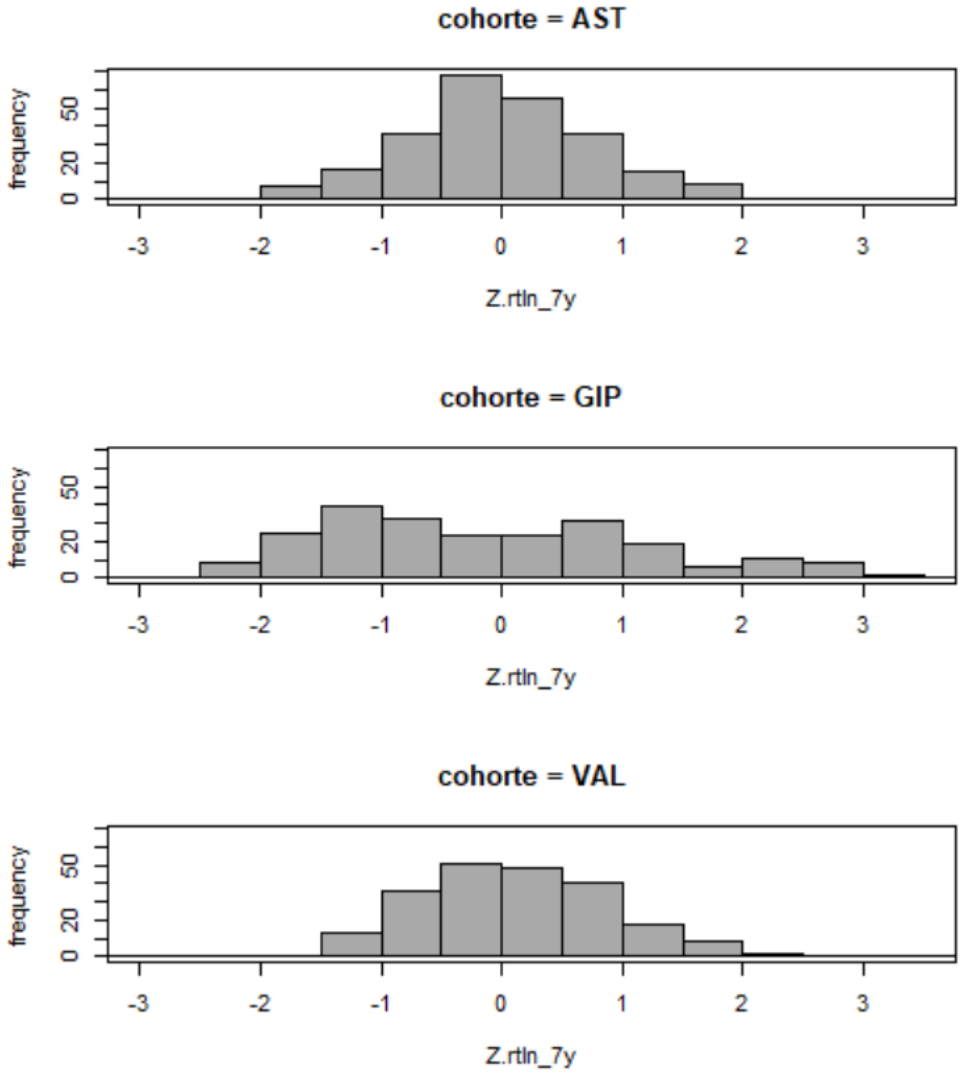

Figure S9. Distribution of leukocyte telomere length ranking change between 4 and 8 years.

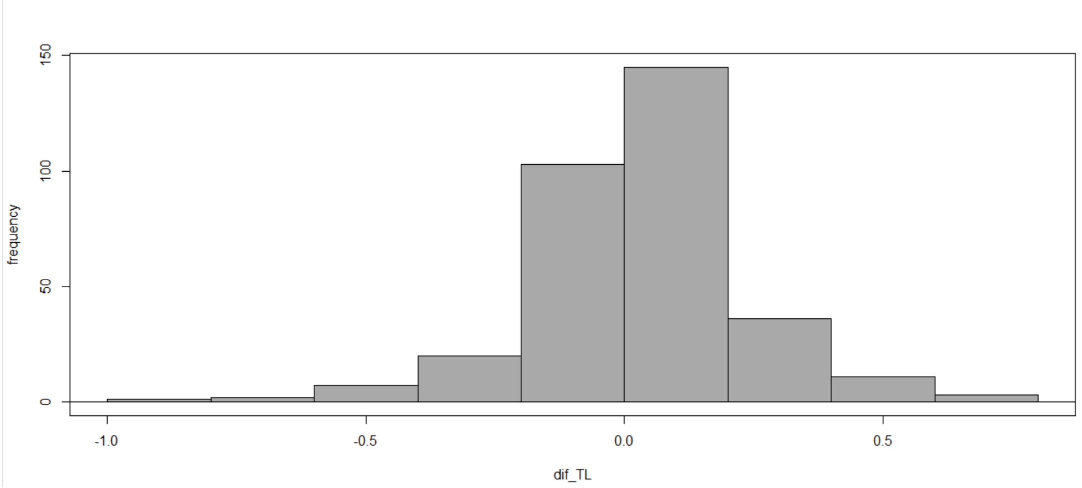

Figure S10. Distribution of leukocyte telomere length ranking change between 4 and 8 years by cohort

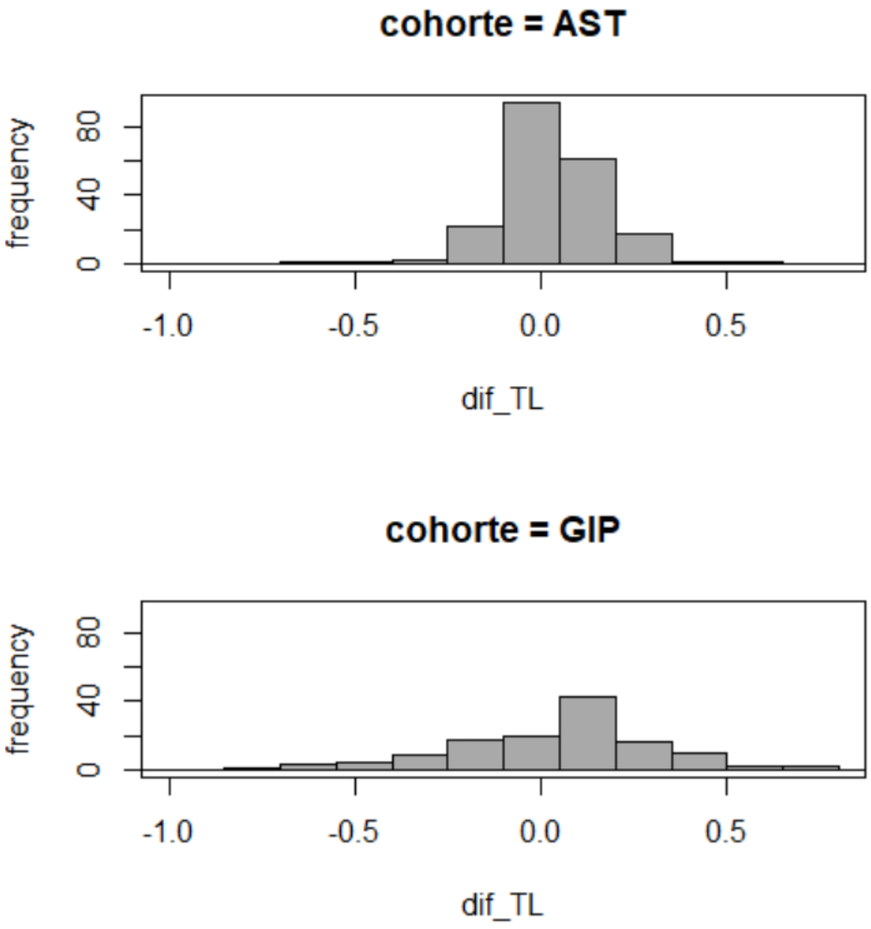

Figure S11. Distribution of z-score leukocyte telomere length ranking change between 4 and 8 years.

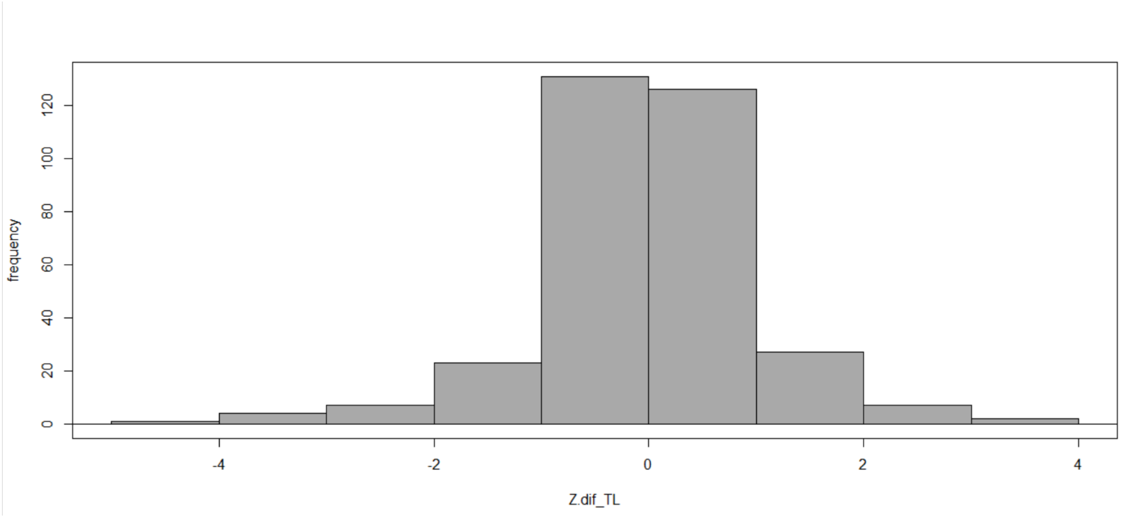

Figure S12. Distribution of z-score leukocyte telomere length ranking change between 4 and 8 years by cohort

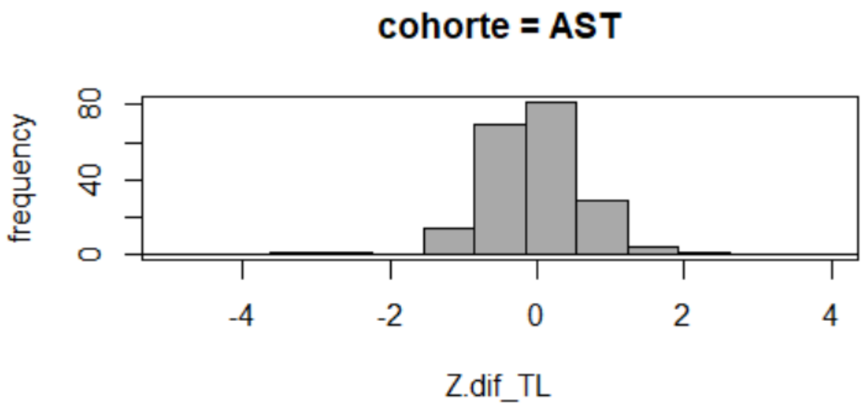

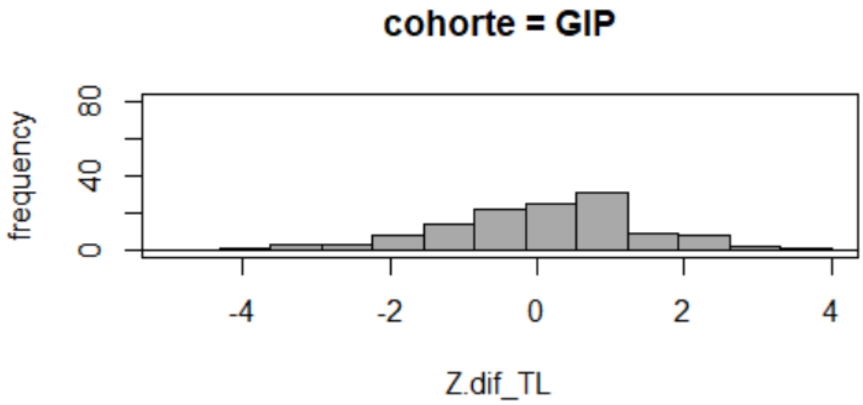

Section S3. Correlation Between Telomere Length at 4 and 8 Years

Figure S13. Correlation between leukocyte telomere length at 4 and 8 years.

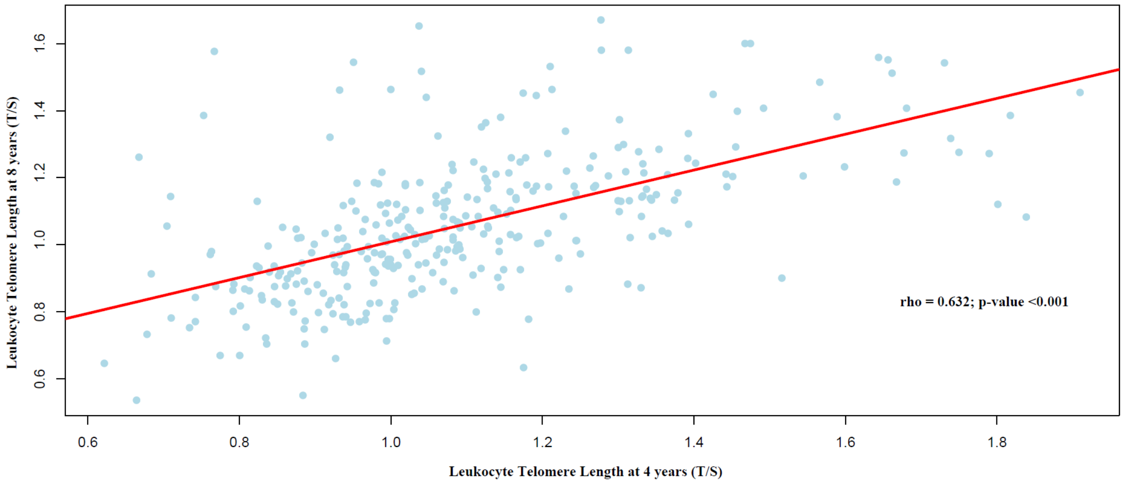

Section S4. Interaction with Child's Sex

P-values are shown for both count and rate components (OR and IRR) from ZINB/NB models. Models adjusted as detailed in footnotes.

Table S1. Interaction between telomere length and child's sex in relation to psychoemotional problems (internalizing, externalizing, and total score).

	**Sex**
**TL at 4 years[a]**	
Internalizing problems	
Count (OR)	0.712
IRR	0.203
Externalizing problems	0.608
Total score	0.011
**Tl at 8 years[b]**	
Internalizing problems	
Count (OR)	0.266
IRR	0.080
Externalizing problems	0.180
Total score	0.061
**TL ranking change from 4 to 8 years[c]**	
Internalizing problems	
Count (OR)	0.978
IRR	0.009
Externalizing problems	0.156
Total score	0.201

[a] Models adjusted by mother's age (continuous), educational level (primary or less, secondary or university), smoking during pregnancy (yes or no), preconceptional body mass index (continuous), child's sex (female or male) and the change values in body mass index and age between 4 to 8 years (continuous).

[b] Models adjusted by mother's age (continuous), educational level (primary or less, secondary or university), smoking during pregnancy (yes or no), preconceptional body mass index (continuous), child's sex (female or male), age (continuous) and body mass index at 8 years (continuous).

[C] Models adjusted by mother's age (continuous), educational level (primary or less, secondary or university), smoking during pregnancy (yes or no), preconceptional body mass index (continuous), child's sex (female or male) and the change values in body mass index and age between 4 to 8 years (continuous) and telomere length at 4 years (basal z-score).
